# Special Issue “Molecular Application of Mass Spectrometry and Chromatography in Biomedicine”

**DOI:** 10.3390/ijms27156659

**Published:** 2026-07-25

**Authors:** Giovanni Ventura, Mariachiara Bianco

**Affiliations:** Dipartimento di Chimica and Centro di Ricerca Interdipartimentale SMART, University of Bari Aldo Moro, Via Orabona 4, 70126 Bari, Italy; mariachiara.bianco@uniba.it

Mass spectrometry (MS) and chromatography have become crucial pillars of biomedical studies. Their coupling, indeed, allows researchers to tackle problems that range from the structural elucidation of biomolecules to their absolute quantification in highly complex biological matrices. The sensitivity, selectivity and versatility of these platforms make them fundamental tools for understanding the molecular mechanisms of disease, for biomarker discovery, for the study of drug metabolism and for the advancement of precision medicine [1,2].

This Special Issue, entitled *Molecular Application of Mass Spectrometry and Chromatography in Biomedicine*, collects eight contributions that exemplify the breadth of these approaches (Table 1). Despite the diversity of the fields addressed, the papers share a common denominator: chromatography and MS are employed as tools to answer concrete biological and clinical questions. The collection can be read along two complementary lines. On the one hand, these techniques act as measurement tools, often replacing established but less reliable methodologies; on the other hand, they act as discovery tools, capable of generating new knowledge on pathological mechanisms and of identifying molecular markers with diagnostic and prognostic value. Furthermore, the eight contributions fall into three thematic groups, namely methodological innovation, clinical bioanalysis, and biomarker discovery, each drawing on these two lines in different proportions.

**Methodological Innovation**. Di Ianni and co-workers propose a liquid chromatography-high resolution mass spectrometry (LC-HRMS) approach for the characterization of antibody–drug conjugates (ADCs), a class of therapeutic proteins central to cancer therapy, in which a cytotoxic payload is delivered selectively to tumor cells by conjugation to a monoclonal antibody [3]. Monitoring the “active” drug-to-antibody ratio (DAR) in circulation is highly informative but analytically demanding [4]. Indeed, payload hydrolysis involves minimal mass shifts that targeted multiple reaction monitoring (MRM) methods may overlook and that even high-resolution analyzers struggle to resolve on the intact antibody. To address this, the authors combine two complementary full-scan MS^2^ strategies that exploit the cleavable nature of the linker to release the payload in the gas phase, reproducing the cleavage that would occur in the lysosome without requiring separate enzymatic or chemical pretreatment. In this manner the small modifications are directly detectable, so that the sites of hydrolysis can be localized and the active DAR refined in both in vitro stability and in vivo pharmacokinetic samples, showing how high resolution coupled to a targeted fragmentation strategy can discriminate what would remain invisible using only MS^1^ analyses.

Lou and co-workers address a longstanding challenge in pharmaceutical quality control: the determination of trace azide, a genotoxic impurity, in angiotensin II receptor blockers (ARBs) [5]. These second-generation antihypertensive drugs are synthesized using azide intermediates, whose residual presence poses significant toxicological concerns even at trace levels, prompting stringent pharmacopoeial limits. However, existing analytical methods often lack sufficient sensitivity or require laborious sample pretreatment. Although ion chromatography is well suited to the determination of this small, highly polar anion, the poor aqueous solubility of ARBs and the resulting matrix interference hinder direct analysis. The authors overcome these limitations through an in-line matrix-elimination strategy based on a two-dimensional column-switching configuration, in which the hydrophobic drug matrix is diverted before reaching the analytical column, thereby enabling direct analysis without pretreatment while achieving detection limits well below pharmacopoeial thresholds. The work illustrates how intelligent fluidic design can resolve a matrix-compatibility problem that would otherwise require off-line sample preparation.

The contribution by Ventura and co-workers belongs to the field of untargeted lipidomics [6] and addresses the structural elucidation of complex lipids, applied to peripheral blood mononuclear cells (PBMCs) of autistic children [7]. Using hydrophilic interaction liquid chromatography (HILIC) coupledwith MS/MS on a linear ion trap, more than two hundred glycerophospholipid and sphingolipid species were characterized, with particular attention to the diagnostic fragmentation patterns useful for determining the regiochemistry of the acyl chains and for distinguishing vinyl ether from ether-linked forms [8,9]. This characterization is further supported by a freely available Excel spreadsheet designed for MS/MS spectra simulation in negative ion mode. Developed as a practical tool, it summarizes the core fragmentation rules of lipid classes and assists users in manual data interpretation, improving the reliability of structural annotation. By providing this tool, the authors promote Open Science principles, enhancing reproducibility and making complex lipid annotation more accessible to the broader scientific community.

**Clinical Bioanalysis**. A second group of papers turns to direct clinical applications, focusing on drug quantification in biological fluids and therapeutic drug monitoring. Nagaoka and co-workers compare the ocular pharmacokinetics of aflibercept and brolucizumab, two anti-VEGF agents of very different size (115 kDa versus 26 kDa), used as intravitreal injections in the treatment of neovascular eye disease. Since the quantification of monoclonal antibodies by enzyme-linked immunosorbent assay ELISA is prone to interference from endogenous ligands and anti-drug antibodies, the authors resort to nano-surface and molecular-orientation limited proteolysis (nSMOL) [10] coupled with LC-MS/MS. Because brolucizumab lacks the Fc domain that the standard nSMOL format relies on for capture, the authors developed a reversed configuration in which the antibody is instead captured via its kappa light chain, making it possible to measure both drugs under the same operating conditions in human aqueous humor. The resulting intraocular half-lives (2.88 days for aflibercept versus 9.00 days for brolucizumab) differ considerably despite the drugs’ comparable clinical indication, a finding with direct relevance to how dosing intervals are set in practice, and illustrate the advantage of MS over immunoassays when the sample matrix contains interfering species. This growing shift toward MS-based platforms is central to precision medicine, because these methods can distinguish structural variants, including specific glycosylation patterns and drug-to-antibody ratios, that conventional immunoassay kits often cannot resolve.

In the same vein is the work of Cecchin and co-workers, devoted to therapeutic drug monitoring in oncology. Monitoring exposure to letrozole and to the cyclin-dependent kinase 4/6 inhibitors (palbociclib, ribociclib and abemaciclib) [11] is clinically motivated by their established exposure-toxicity relationships and by the value of tracking treatment adherence over long courses of therapy, yet routine monitoring has so far been limited by the reliance on venous plasma sampling. The authors develop and validate an LC-MS/MS method for the quantification of these drugs and their metabolites in volumetric dried blood spots, collected with a dedicated microsampling device. The assay proved independent of hematocrit, a variable that commonly biases dried blood spot methods, and the authors also built conversion models to estimate the plasma concentrations still used as the clinical reference for dosing decisions. Paper-based sampling is in any case far simpler to collect and ship than venous blood, which is what makes a decentralized, less invasive monitoring scheme realistic for patients under long-term treatment for breast cancer.

Franco, Palmisani and co-workers present an LC-MS/MS method for the determination of ambroxol. Ambroxol, originally used as a mucolytic agent, is now studied as a pharmacological chaperone capable of crossing the blood–brain barrier. It enhances glucocerebrosidase activity and reduces α-synuclein levels, making it a promising candidate for disease-modifying therapy in Parkinson’s disease associated with mutations in the GBA1 gene [12]. Assessing whether the drug reaches the brain requires measuring it directly in cerebrospinal fluid, in addition to plasma. Compared with the previous reference method, the new assay requires only one-fifth of the sample volume, thanks to an online solid-phase extraction that also removes the evaporation step altogether. An extensive stability assessment, covering storage at different temperatures, freeze–thaw cycles and prolonged time on the autosampler, gives the method the robustness required by multicenter sample logistics. Validated according to European Medicines Agency (EMA) guidelines, the assay was applied within the AMBITIOUS phase II trial, where it confirmed brain penetration directly in patients, illustrating how a robust bioanalytical method underpins clinical investigation.

**Biomarker Discovery**. A third group of contributions is oriented towards biomarker discovery and the understanding of pathological mechanisms. Distinct from the preceding contributions, the review by Lella and co-workers steps back from a single method or matrix to survey the whole emerging field of D-amino acids as biomarkers of neurological disease [13], bringing together the pharmacological rationale and the analytical toolbox in a single narrative. On the biological side, the review traces how fluctuations in D-amino acid levels, through their modulation of N-methyl-D-aspartate receptors and neurotransmission, have been associated with pathological states as diverse as schizophrenia, Alzheimer’s disease, epilepsy and Parkinson’s disease. On the analytical side, it maps out why these findings have been so hard to establish. D- and L-amino acids are indistinguishable by standard achiral LC-MS/MS, and the challenge is compounded by the D-form typically being present in trace amounts against a large excess of its L counterpart. Derivatization with a chiral reagent, converting the enantiomers into diastereomers that a conventional column can resolve, emerges from the review as the strategy of choice for this task. By consolidating findings scattered across disease models and analytical platforms, the review offers a reference point for a field that has so far lacked one.

Finally, Váradi investigates the glycosylation of serum immunoglobulin G in post-COVID-19 and post-vaccination patients, using HILIC coupled with fluorescence and MS detection (HILIC-FLR-MS). The analysis of the IgG N-glycome, carried out after PNGase F digestion and procainamide labeling, reveals higher levels of sialylation and afucosylation in patients who contracted the infection, with a further increase induced by vaccination limited to previously infected subjects. Since terminal sialylation is associated with the anti-inflammatory activity of IgGs [14] and modulates binding to the FcγRIIIa receptor, these findings help to clarify the relationship between the glycosylation pattern and the immune response and suggest that natural infection may elicit a more pronounced glycosylation shift than vaccination alone.

Seen together, the eight contributions align with the two lines introduced earlier. As measurement tools, chromatography and mass spectrometry increasingly serve as a reference standard where conventional methods reach their limits in complex biological or pharmaceutical matrices, whether by replacing immunoassays prone to interference or by resolving matrix incompatibilities that make standard chromatographic approaches inapplicable. The same logic drives the growing use of microsampling, which opens the way to decentralized, minimally invasive therapeutic drug monitoring.

As discovery tools, chromatography and mass spectrometry underpin the structural characterization of complex biomolecules, together with the annotation tools that make the resulting data interpretable, letting ever finer differences become visible and eventually informative. The same capability drives the search for biomarkers in neurodegenerative disease, where the trace levels or the chirality of the target analytes place demands on sensitivity and selectivity that LC-MS/MS is especially well suited to meet. Ultimately, these two roles, measurement and discovery, are what make chromatography and mass spectrometry increasingly indispensable for clinical diagnostics and molecular medicine. Looking ahead, the integration of Artificial Intelligence and Machine Learning is likely to become a key frontier for interpreting the large datasets generated by these platforms, especially in the untargeted lipidomics and biomarker discovery studies discussed here.

## Figures and Tables

**Table 1:** List of the articles present in this Special Issue.

First Author	Reference
Di Ianni, A.	Di Ianni, A.; Cowan, K.J.; Riccardi Sirtori, F.; Barbero, L. *Unlocking the Complexity of Antibody-Drug Conjugates: A Cutting Edge LC-HRMS Approach to Refine Drug-to-Antibody Ratio Measurements with Highly Reactive Payloads*. Int. J. Mol. Sci. 2025, 26, 3080, doi:10.3390/ijms26073080.
Lou, C.	Lou, C.; Pan, S.; Yu, X.; Zhang, K.; Zhang, K.; Zhu, Y. An *Automated and Precise Approach for the Determination of Azide Residue in Angiotensin II Receptor Blockers Using* In Situ *Matrix Elimination Ion Chromatography with Switching Strategy.* Int. J. Mol. Sci. 2025, 26, 4895, doi:10.3390/ijms26104895.
Ventura, G.	Ventura, G.; Bianco, M.; Calvano, C.D.; Losito, I.; Cataldi, T.R.I. *Tandem Mass Spectrometry in Untargeted Lipidomics: A Case Study of Peripheral Blood Mononuclear Cells.* Int. J. Mol. Sci. 2024, 25, 12077, doi:10.3390/ijms252212077.
Nagaoka, K.	Nagaoka, K.; Kimura, N.; Inoda, S.; Takayama, T.; Arai, Y.; Yanagi, Y.; Shimada, T.; Nagai, R.; Takahashi, H.; Aizawa, K. *Comparative Pharmacokinetic Analysis of Aflibercept and Brolucizumab in Human Aqueous Humor Using Nano-Surface and Molecular-Orientation Limited Proteolysis.* Int. J. Mol. Sci. 2025, 26, 556, doi:10.3390/ijms26020556.
Cecchin, E.	Cecchin, E.; Orleni, M.; Gagno, S.; Montico, M.; Peruzzi, E.; Roncato, R.; Gerratana, L.; Corsetti, S.; Puglisi, F.; Toffoli, G.; et al. *Quantification of Letrozole, Palbociclib, Ribociclib, Abemaciclib, and Metabolites in Volumetric Dried Blood Spots: Development and Validation of an LC-MS/MS Method for Therapeutic Drug Monitoring.* Int. J. Mol. Sci. 2024, 25, 10453, doi:10.3390/ijms251910453.
Franco, V.; Palmisani, M.	Franco, V.; Palmisani, M.; Colucci, F.; De Micco, R.; Aloisio, S.; Cazzaniga, F.; Cerri, S.; Crema, F.; Dattrino, F.; Garavaglia, B.; et al. *LC-MS/MS-Based Determination of Ambroxol in Human Plasma and Cerebrospinal Fluid: Validation and Applicability in a Phase II Study on GBA-Associated Parkinson’s Disease Patients.* Int. J. Mol. Sci. 2025, 26, 6094, doi:10.3390/ijms26136094.
Lella, C.	Lella, C.; Nestor, L.; De Bundel, D.; Vander Heyden, Y.; Van Eeckhaut, A. *Targeted Chiral Metabolomics of D-Amino Acids: Their Emerging Role as Potential Biomarkers in Neurological Diseases with a Focus on Their Liquid Chromatography–Mass Spectrometry Analysis upon Chiral Derivatization.* Int. J. Mol. Sci. 2024, 25, 12410, doi:10.3390/ijms252212410.
Váradi, C.	Váradi, C. *The Glycosylation of Serum IgG Antibodies in Post-COVID-19 and Post-Vaccination Patients.* Int. J. Mol. Sci. 2025, 26, 807, doi:10.3390/ijms26020807.

## References

[1] Son A., Kim W., Park J., Park Y., Lee W., Lee S., Kim H. (2024). Mass Spectrometry Advancements and Applications for Biomarker Discovery, Diagnostic Innovations, and Personalized Medicine. Int. J. Mol. Sci..

[2] Thomas S.N., French D., Jannetto P.J., Rappold B.A., Clarke W.A. (2022). Liquid Chromatography–Tandem Mass Spectrometry for Clinical Diagnostics. Nat. Rev. Methods Prim..

[3] Beck A., Goetsch L., Dumontet C., Corvaïa N. (2017). Strategies and Challenges for the next Generation of Antibody-Drug Conjugates. Nat. Rev. Drug Discov..

[4] Ieki K., Fukuda S., Miyawaki S., Hirowatari K. (2025). Regulated Bioanalysis of Antibody-Drug Conjugates Using LC-MS. Bioanalysis.

[5] Michel M.C., Foster C., Brunner H.R., Liu L. (2013). A Systematic Comparison of the Properties of Clinically Used Angiotensin II Type 1 Receptor Antagonists. Pharmacol. Rev..

[6] Hsu F.F., Turk J. (2007). Differentiation of 1-O-Alk-1′-Enyl-2-Acyl and 1-O-Alkyl-2-Acyl Glycerophospholipids by Multiple-Stage Linear Ion-Trap Mass Spectrometry with Electrospray Ionization. J. Am. Soc. Mass Spectrom..

[7] Ventura G., Calvano C.D., Porcelli V., Palmieri L., De Giacomo A., Xu Y., Goodacre R., Palmisano F., Cataldi T.R.I.I. (2020). Phospholipidomics of Peripheral Blood Mononuclear Cells (PBMCs): The Tricky Case of Children with Autism Spectrum Disorder (ASD) and Their Healthy Siblings. Anal. Bioanal. Chem..

[8] Hsu F.-F., Turk J. (2009). Electrospray Ionization with Low-Energy Collisionally Activated Dissociation Tandem Mass Spectrometry of Glycerophospholipids: Mechanisms of Fragmentation and Structural Characterization. J. Chromatogr. B.

[9] Farooqui A.A., Horrocks L.A. (2001). Plasmalogens: Workhorse Lipids of Membranes in Normal and Injured Neurons and Glia. Neuroscientist.

[10] Iwamoto N., Shimada T., Umino Y., Aoki C., Aoki Y., Sato T.-A., Hamada A., Nakagama H. (2014). Selective Detection of Complementarity-Determining Regions of Monoclonal Antibody by Limiting Protease Access to the Substrate: Nano-Surface and Molecular-Orientation Limited Proteolysis. Analyst.

[11] Onesti C.E., Jerusalem G. (2021). CDK4/6 Inhibitors in Breast Cancer: Differences in Toxicity Profiles and Impact on Agent Choice. A Systematic Review and Meta-Analysis. Expert Rev. Anticancer Ther..

[12] Vieira S.R.L., Schapira A.H.V. (2022). Glucocerebrosidase Mutations and Parkinson Disease. J. Neural Transm..

[13] Wolosker H., Dumin E., Balan L., Foltyn V.N. (2008). D-Amino Acids in the Brain: D-Serine in Neurotransmission and Neurodegeneration. FEBS J..

[14] Kaneko Y., Nimmerjahn F., Ravetch J.V. (2006). Anti-Inflammatory Activity of Immunoglobulin G Resulting from Fc Sialylation. Science.

